# 2-{[2,2-Bis(diethyl­amino)­ethan-2-ylium­thio­yl]sulfan­yl}-1,1-bis­(diethyl­amino)­ethyl­ium bis­(perchlorate)

**DOI:** 10.1107/S1600536812035453

**Published:** 2012-08-23

**Authors:** Keiji Ohno, Tomoaki Sugaya, Takashi Fujihara, Akira Nagasawa

**Affiliations:** a Department of Chemistry, Graduate School of Science and Engineering , Saitama University, Shimo-Okubo 255, Sakura-ku, Saitama 338-8570, Japan; b Comprehensive Analysis Center for Science, Saitama University, Shimo-Okubo 255, Sakura-ku, Saitama 338-8570, Japan

## Abstract

The title salt, C_20_H_42_N_4_S_2_^2+^·2ClO_4_^−^, was obtained from the reaction of bis­(diethyl­amino)­carbeniumdithio­carboxyl­ate, (Et_2_N)_2_C_2_S_2_, with Fe(ClO_4_)_2_·6H_2_O in CH_2_Cl_2_. The title compound, in which one of the S atoms of (Et_2_N)_2_C_2_S_2_ is bound to a 1,1-bis­(diethyl­amino)­ethane moiety, has two carbenium C atoms, and the charge compensation is provided by two perchlorate anions. The N_2_C—CS_2_ bond length is 1.512 (4) Å, corresponding to a C—C single bond, and the dihedral angle between N_2_C– and –CS_2_ planes [72.0 (2)°] is smaller than that of (Et_2_N)_2_C_2_S_2_ [82.0 (1)°]. The crystal structure features C—H⋯S hydrogen bonds.

## Related literature

For general background to bis­(*N*,*N*-disubstituted amino)­carbeniumdithio­carboxyl­ates, see: Winberg & Coffman (1965 ▶); Nagasawa *et al.* (1995 ▶); Nakayama & Akiyama (1992 ▶); Nakayama *et al.* (1997 ▶, 2000 ▶); Nakayama (2000 ▶, 2002 ▶); Miller *et al.* (2000 ▶); Fujihara *et al.* (2002 ▶); Siemeling *et al.* (2012 ▶). For transition metal complexes, see: Miyashita *et al.* (1998 ▶); Banerjee *et al.* (2002 ▶); Fujihara *et al.* (2004 ▶); Sugaya *et al.* (2009 ▶). For the cationic dimer of bis­(*N*,*N*-disubstituted amino)­carbeniumdithio­carboxyl­ates, see: Otani *et al.* (1998 ▶); Banerjee & Zubieta (2004 ▶). For reference bond-length data, see: Allen *et al.* (1987 ▶).
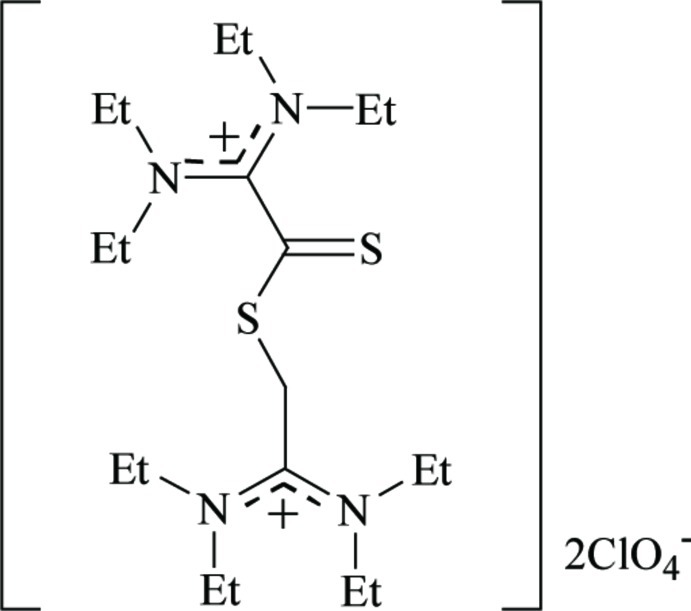


## Experimental

### 

#### Crystal data

C_20_H_42_N_4_S_2_^2+^·2ClO_4_^−^
*M* *_r_* = 601.62
Orthorhombic, 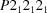

*a* = 8.4158 (7) Å
*b* = 16.1889 (13) Å
*c* = 21.7213 (18) Å
*V* = 2959.4 (4) Å^3^
*Z* = 4
Mo *K*α radiation
μ = 0.41 mm^−1^
*T* = 150 K
0.32 × 0.21 × 0.20 mm

#### Data collection

Bruker SMART APEX CCD diffractometer
Absorption correction: multi-scan (*SADABS*; Sheldrick, 1996 ▶) *T*_min_ = 0.881, *T*_max_ = 0.923
21803 measured reflections
7058 independent reflections
5641 reflections with *I* > 2σ(*I*)
*R*_int_ = 0.046

#### Refinement

*R*[*F* ^2^ > 2σ(*F* ^2^)] = 0.055
*wR*(*F* ^2^) = 0.140
*S* = 1.02
7058 reflections
333 parameters
H-atom parameters constrained
Δρ_max_ = 0.70 e Å^−3^
Δρ_min_ = −0.26 e Å^−3^
Absolute structure: Flack (1983 ▶), 3081 Friedel pairs
Flack parameter: 0.00 (7)

### 

Data collection: *SMART* (Bruker, 2003 ▶); cell refinement: *SAINT* (Bruker, 2003 ▶); data reduction: *SAINT*; program(s) used to solve structure: *SHELXTL*; program(s) used to refine structure: *SHELXTL*; molecular graphics: *ORTEP-3* (Farrugia, 1997 ▶); software used to prepare material for publication: *SHELXTL* (Sheldrick, 2008 ▶).

## Supplementary Material

Crystal structure: contains datablock(s) global, I. DOI: 10.1107/S1600536812035453/rk2371sup1.cif

Structure factors: contains datablock(s) I. DOI: 10.1107/S1600536812035453/rk2371Isup2.hkl

Additional supplementary materials:  crystallographic information; 3D view; checkCIF report

## Figures and Tables

**Table 1:** Hydrogen-bond geometry (Å, °)

*D*—H⋯*A*	*D*—H	H⋯*A*	*D*⋯*A*	*D*—H⋯*A*
C14—H14*B*⋯S1^i^	0.98	2.91	3.893 (4)	177

Symmetry code: (i) 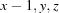.

**Fig. 1.**
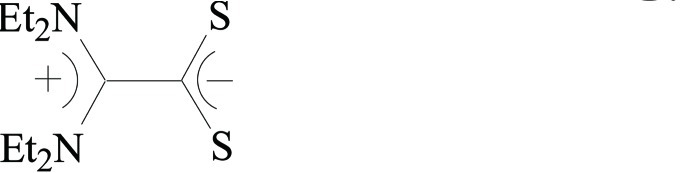
The chemical structure of (II).

**Fig. 2.**
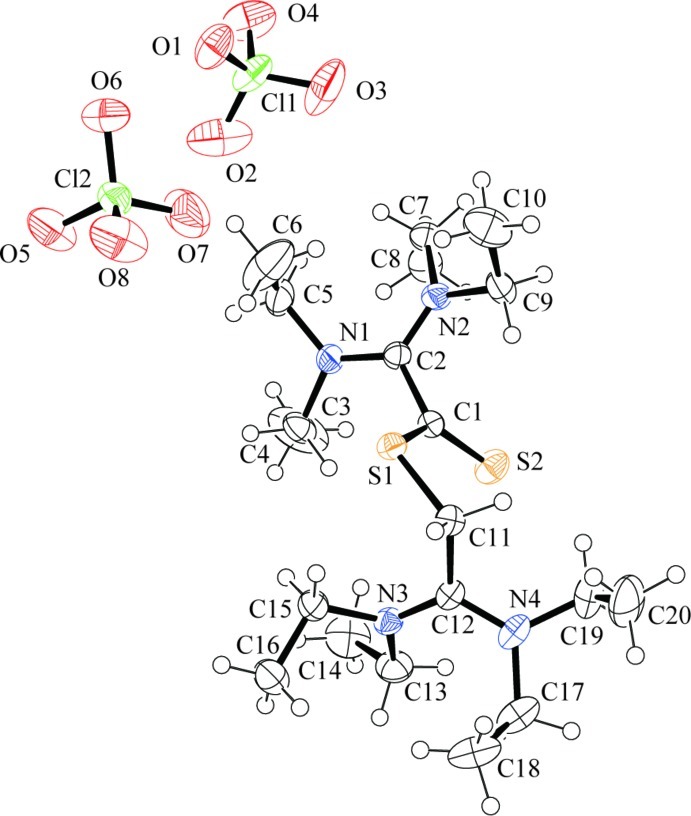
The molecular structure of I with the atom numbering scheme. Displacement ellipsoids are drawn at the 50% probability level. H atoms are presented as a small spheres of arbitrary radius.

**Fig. 3.**
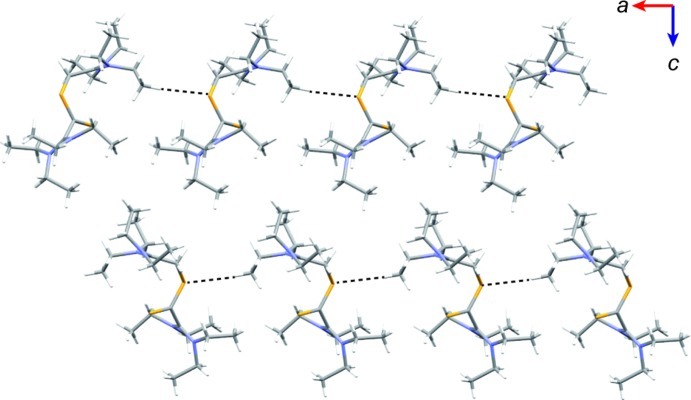
A view of the crystal packing, showing the hydrogen bonding (dashed lines). All counter ions (ClO4-) have been omitted for clarity.

## supplementary crystallographic information

### Comment

Bis(*N*,*N*-disubsituted amino)carbeniumdithiocarboxylates [(R_2_N)_2_C_2_S_2_], which have a zwitterionic (inner-salt) form and a neutral form as canonical structures (Nagasawa *et al.*, 1995; Fujihara *et al.*, 2002), are structurally and reactively interesting (Winberg & Coffman, 1965; Otani *et al.*, 1998; Nakayama & Akiyama, 1992; Nakayama *et al.*, 2000; Nakayama, 2000, 2002; Banerjee & Zubieta, 2004; Siemeling *et al.*, 2012). We have reported the syntheses, structures, and properties of the various transition metal complexes using (R_2_N)_2_C_2_S_2_ as ligands (Miyashita *et al.*, 1998; Banerjee *et al.*, 2002; Fujihara *et al.*, 2004; Sugaya *et al.*, 2009), and the ligands act as neutral monodentate, bridging, and chelating ligands maintaining the characteristic zwitterionic form.

In the process of a preparation of iron(II) complex with bis(*N*,*N*-diethylamino)carbeniumdithiocarboxylate [(Et_2_N)_2_C_2_S_2_] (II) (Fig 1), we obtained the unexpectedly bis(diethylamino)-methylium bis(diethylamino)-2-ethyliumcarbodithioate perchlorate [(C_10_H_20_N_2_S_2_)(C_10_H_20_N_2_)](ClO_4_)_2_ (I) and report here its molecular structure. We suppose that I was formed through an oxidation of II, which has taken place under gentle conditions for several months in solution. The molecular structure of I is shown in Fig. 2. The C11–S1 bond length is 1.794 (3) Å, which corresponds to the C–S single bond [1.79 (1)–1.82 (1)Å; Miller *et al.*, 2000; 1.78 (4)Å; Nakayama *et al.*, 1997]. The N–C(CS_2_) bond lengths in the range of 1.316 (4)–1.333 (4) Å are slightly shorter than the normal C(*sp*^3^)–N(*sp*^3^) bond length (1.36Å; Allen *et al.*, 1987) suggesting that the C2 and C12 atoms are carbenium carbons. The bond lengths of C1–S2 [1.615 (3) Å] and C1–S1 [1.729 (3) Å] are close to those of the CS terminal and the –C–S– bridging bonds of methylated species of I, respectively [1.608 (14)Å and 1.714 (13)Å, respectively; Nakayama *et al.*, 1997], indicating localization of electron on S–C–S moiety. The C1–C2 bond length [1.512 (4) Å] and dihedral angle between N_2_C– and –CS_2_ planes [71.99 (22)°] are slightly longer and smaller, respectively, than those of II [1.477Å–1.506 (2)Å and 82.0 (1)°, respectively; Nagasawa *et al.*, 1995], and these change mean that decrease of interaction between the unfilled *p* orbital of carbenium carbon (C2) and electrons on S–C–S moiety. The C11–C12 bond length 1.523 (4)Å is slightly longer than that of C1–C2. The S1–C1–S2, N1–C2–N2, and N3–C12–N4 bond angles are similar values for those of II [S–C–S: 129.4 (8)° and N–C–N: 122 (1)°; Nagasawa *et al.*, 1995]. Two ClO_4_^-^ per one I exist as counter ions in the crystal. The crystal structure consists of a chain structure through intermolecular weak C—H···S hydrogen bonding [H14B···S1': 2.9138 Å, C14–H14B···S1': 176.69°] (Fig. 3 and Hydrogen-bond geometry).

### Experimental

All the processes were carried out under an argon atmosphere using standard Schlenk techniques. Fe(ClO_4_)_2_^.^6H_2_O (0.076 g, 0.21 mmol) was dissolved in CH_3_CN (15 cm^3^). After stirring for 1 h at room temperature, the solvent was removed by evaporation under reduced pressure. The resulting powder (white) and II (0.141 g, 0.60 mmol) were dissolved in CH_2_Cl_2_ (30 cm^3^) and stirred for 1 h at room temperature. The insoluble salt was then filtered off, and the solvent of the filtrate was removed by evaporation under reduced pressure. The resulting powder was dissolved in CH_2_Cl_2_, layered with Et_2_O, and set aside for several months at room temperature. The red-purple crystals were obtained and dried *in vacuo*. Yield 0.011 g. (6.2% based on the II). ^1^H NMR, CD_3_CN *δ* 4.62 (*s*, 2H, -S–C*H_2_*-), 3.55 (*dq*, 16H, CH_3_–C*H_2_*-) 1.27 (*q*, 24H, C*H_3_*-). ^13^C NMR, CD_3_CN *δ* 213.3 (S-*C*-S), 167.0 (N–*C*–N), 163.6 (N–*C*–N), 49.1 (CH_3_–*C*H_2_-), 48.7 (CH_3_–*C*H_2_-), 36.9 (S-*C*H_2_-), 13.7 (*C*H_3_). Analysis found: C 39.72, H 7.04, N 9.16%; calculated for C_20_H_42_Cl_2_N_4_O_8_S_2_: C 39.93, H 7.04, N 9.31%.

### Refinement

All the non-hydrogen atoms were refined anisotropically. All H atoms were placed in geometrically idealized positions and treated as riding on their parent atoms with C–H = 0.99Å, *U*_iso_(H) = 1.2*U*_eq_(C) for methylene atoms and C–H = 0.98Å, *U*_iso_(H) = 1.5*U*_eq_(C) for methyl atoms.

### Crystal data

**Table:**

C_20_H_42_N_4_S_2_^2+^·2ClO_4_^−^	*F*(000) = 1280
*M**_r_* = 601.62	*D*_x_ = 1.350 Mg m^−^^3^
Orthorhombic, *P*2_1_2_1_2_1_	Mo *K*α radiation, λ = 0.71073 Å
Hall symbol: P 2ac 2ab	Cell parameters from 3791 reflections
*a* = 8.4158 (7) Å	θ = 2.3–25.2°
*b* = 16.1889 (13) Å	µ = 0.41 mm^−^^1^
*c* = 21.7213 (18) Å	*T* = 150 K
*V* = 2959.4 (4) Å^3^	Block, red
*Z* = 4	0.32 × 0.21 × 0.20 mm

### Data collection

**Table:**

Bruker SMART APEX CCD diffractometer	7058 independent reflections
Radiation source: fine-focus sealed tube	5641 reflections with *I* > 2σ(*I*)
Graphite monochromator	*R*_int_ = 0.046
Detector resolution: 8.366 pixels mm^-1^	θ_max_ = 27.9°, θ_min_ = 1.6°
φ and ω scans	*h* = −11→11
Absorption correction: multi-scan (*SADABS*; Sheldrick, 1996)	*k* = −20→21
*T*_min_ = 0.881, *T*_max_ = 0.923	*l* = −18→28
21803 measured reflections	

### Refinement

**Table:**

Refinement on *F*^2^	Secondary atom site location: difference Fourier map
Least-squares matrix: full	Hydrogen site location: inferred from neighbouring sites
*R*[*F*^2^ > 2σ(*F*^2^)] = 0.055	H-atom parameters constrained
*wR*(*F*^2^) = 0.140	*w* = 1/[σ^2^(*F*_o_^2^) + (0.0814*P*)^2^*P*] where *P* = (*F*_o_^2^ + 2*F*_c_^2^)/3
*S* = 1.02	(Δ/σ)_max_ = 0.003
7058 reflections	Δρ_max_ = 0.70 e Å^−^^3^
333 parameters	Δρ_min_ = −0.26 e Å^−^^3^
0 restraints	Absolute structure: Flack (1983), 3081 Friedel pairs
Primary atom site location: structure-invariant direct methods	Flack parameter: 0.00 (7)

### Special details

**Table:**

Geometry. Least-squares planes (*x*,*y*,*z* in crystal coordinates) and deviations from them (* indicates atom used to define plane)- 6.5375 (0.0177) *x* + 3.5865 (0.0590) *y* + 12.8042 (0.0841) *z* = 5.1962 (0.0328)* 0.0000 (0.0000) N1 * 0.0000 (0.0000) C2 * 0.0000 (0.0000) N2Rms deviation of fitted atoms = 0.00006.1258 (0.0105) *x* + 10.6803 (0.0361) *y* + 4.0595 (0.0680) *z* = 11.1638 (0.0232)Angle to previous plane (with approximate e.s.d.) = 71.99 (0.22)* 0.0000 (0.0000) S1 * 0.0000 (0.0000) C1 * 0.0000 (0.0000) S2Rms deviation of fitted atoms = 0.0000

### Fractional atomic coordinates and isotropic or equivalent isotropic displacement parameters (Å^2^)

**Table:**

	*x*	*y*	*z*	*U*_iso_*/*U*_eq_	
C1	0.0803 (3)	0.92906 (17)	0.18456 (13)	0.0266 (6)	
C2	0.1428 (3)	0.87247 (17)	0.23435 (13)	0.0275 (6)	
C3	−0.0518 (4)	0.7694 (2)	0.20201 (15)	0.0402 (8)	
H3A	−0.0328	0.7193	0.1769	0.048*	
H3B	−0.0864	0.8142	0.1741	0.048*	
C4	−0.1802 (6)	0.7523 (4)	0.2484 (2)	0.0905 (19)	
H4A	−0.1428	0.7108	0.2779	0.136*	
H4B	−0.2750	0.7315	0.2272	0.136*	
H4C	−0.2066	0.8033	0.2703	0.136*	
C5	0.1951 (5)	0.7249 (2)	0.25762 (18)	0.0510 (9)	
H5A	0.1389	0.6720	0.2506	0.061*	
H5B	0.2093	0.7319	0.3026	0.061*	
C6	0.3555 (7)	0.7219 (3)	0.2270 (2)	0.0891 (19)	
H6A	0.3418	0.7134	0.1826	0.134*	
H6B	0.4176	0.6762	0.2443	0.134*	
H6C	0.4116	0.7741	0.2341	0.134*	
C7	0.2630 (4)	0.8720 (2)	0.33875 (14)	0.0381 (8)	
H7A	0.3331	0.9100	0.3619	0.046*	
H7B	0.3157	0.8174	0.3369	0.046*	
C8	0.1063 (5)	0.8636 (2)	0.37214 (16)	0.0456 (8)	
H8A	0.0482	0.9160	0.3698	0.068*	
H8B	0.1260	0.8496	0.4154	0.068*	
H8C	0.0432	0.8198	0.3529	0.068*	
C9	0.3319 (4)	0.98111 (19)	0.26184 (16)	0.0378 (7)	
H9A	0.3147	1.0217	0.2953	0.045*	
H9B	0.2930	1.0058	0.2230	0.045*	
C10	0.5082 (5)	0.9627 (3)	0.2561 (2)	0.0628 (11)	
H10A	0.5488	0.9430	0.2958	0.094*	
H10B	0.5649	1.0131	0.2442	0.094*	
H10C	0.5247	0.9201	0.2248	0.094*	
C11	0.1161 (3)	0.98670 (17)	0.06450 (13)	0.0274 (6)	
H11A	0.1500	1.0391	0.0841	0.033*	
H11B	0.1794	0.9803	0.0264	0.033*	
C12	−0.0572 (3)	0.99655 (17)	0.04567 (12)	0.0258 (6)	
C13	−0.3217 (4)	0.9369 (2)	0.04633 (17)	0.0427 (8)	
H13A	−0.3786	0.9335	0.0065	0.051*	
H13B	−0.3464	0.9911	0.0652	0.051*	
C14	−0.3781 (5)	0.8689 (3)	0.0880 (2)	0.0585 (11)	
H14A	−0.3607	0.8154	0.0680	0.088*	
H14B	−0.4917	0.8761	0.0964	0.088*	
H14C	−0.3187	0.8708	0.1268	0.088*	
C15	−0.0855 (4)	0.85068 (19)	0.01460 (15)	0.0334 (7)	
H15A	−0.1061	0.8083	0.0465	0.040*	
H15B	0.0309	0.8551	0.0090	0.040*	
C16	−0.1614 (5)	0.8243 (2)	−0.04533 (17)	0.0479 (9)	
H16A	−0.2754	0.8154	−0.0390	0.072*	
H16B	−0.1123	0.7728	−0.0595	0.072*	
H16C	−0.1457	0.8675	−0.0763	0.072*	
C17	−0.2247 (5)	1.0945 (3)	−0.01183 (18)	0.0527 (10)	
H17A	−0.2348	1.1554	−0.0140	0.063*	
H17B	−0.3297	1.0719	−0.0001	0.063*	
C18	−0.1813 (5)	1.0624 (3)	−0.07407 (18)	0.0593 (11)	
H18A	−0.0680	1.0720	−0.0816	0.089*	
H18B	−0.2440	1.0912	−0.1055	0.089*	
H18C	−0.2035	1.0031	−0.0760	0.089*	
C19	−0.0413 (5)	1.1461 (2)	0.06844 (18)	0.0482 (9)	
H19A	0.0134	1.1278	0.1064	0.058*	
H19B	−0.1296	1.1830	0.0807	0.058*	
C20	0.0738 (6)	1.1938 (2)	0.0293 (2)	0.0675 (13)	
H20A	0.1609	1.1574	0.0166	0.101*	
H20B	0.1165	1.2402	0.0530	0.101*	
H20C	0.0188	1.2148	−0.0073	0.101*	
N1	0.0976 (3)	0.79387 (15)	0.23329 (12)	0.0338 (6)	
N2	0.2405 (3)	0.90405 (16)	0.27537 (11)	0.0304 (5)	
N3	−0.1489 (3)	0.93085 (14)	0.03540 (12)	0.0282 (5)	
N4	−0.1076 (3)	1.07317 (15)	0.03665 (13)	0.0372 (7)	
O1	0.6764 (4)	0.61374 (17)	0.34864 (12)	0.0564 (7)	
O2	0.4000 (4)	0.6153 (3)	0.35918 (17)	0.0945 (12)	
O3	0.5508 (5)	0.73360 (19)	0.37979 (16)	0.0918 (12)	
O4	0.5645 (4)	0.6189 (2)	0.44602 (13)	0.0710 (9)	
O5	0.6992 (4)	0.53757 (17)	0.07763 (13)	0.0663 (9)	
O6	0.9442 (4)	0.58632 (19)	0.11349 (17)	0.0749 (9)	
O7	0.7235 (4)	0.6719 (2)	0.11376 (18)	0.0878 (11)	
O8	0.8378 (4)	0.6359 (2)	0.02204 (14)	0.0705 (9)	
Cl1	0.54701 (11)	0.64601 (6)	0.38326 (4)	0.0490 (2)	
Cl2	0.80215 (10)	0.60677 (5)	0.08213 (4)	0.0389 (2)	
S1	0.16983 (8)	0.90400 (4)	0.11539 (3)	0.02782 (16)	
S2	−0.05125 (11)	0.99911 (6)	0.19878 (4)	0.0407 (2)	

### Atomic displacement parameters (Å^2^)

**Table:**

	*U*^11^	*U*^22^	*U*^33^	*U*^12^	*U*^13^	*U*^23^
C1	0.0269 (15)	0.0247 (14)	0.0283 (14)	−0.0014 (11)	−0.0026 (12)	−0.0001 (12)
C2	0.0311 (15)	0.0273 (14)	0.0242 (14)	0.0008 (12)	−0.0004 (12)	0.0014 (12)
C3	0.0454 (19)	0.0397 (17)	0.0354 (17)	−0.0164 (16)	−0.0078 (15)	0.0043 (15)
C4	0.072 (3)	0.147 (5)	0.053 (3)	−0.055 (4)	0.005 (3)	0.008 (3)
C5	0.078 (3)	0.0277 (16)	0.047 (2)	0.0047 (18)	−0.012 (2)	0.0011 (15)
C6	0.121 (5)	0.085 (3)	0.061 (3)	0.061 (3)	0.021 (3)	0.016 (3)
C7	0.0467 (19)	0.0410 (18)	0.0266 (15)	0.0019 (15)	−0.0076 (14)	0.0004 (14)
C8	0.060 (2)	0.0443 (19)	0.0328 (18)	−0.0042 (17)	0.0051 (16)	0.0030 (16)
C9	0.0430 (18)	0.0341 (16)	0.0362 (17)	−0.0109 (15)	−0.0032 (15)	−0.0036 (14)
C10	0.046 (2)	0.083 (3)	0.059 (3)	−0.014 (2)	0.0002 (19)	0.000 (2)
C11	0.0263 (14)	0.0280 (14)	0.0279 (15)	−0.0055 (11)	0.0043 (11)	0.0046 (12)
C12	0.0285 (14)	0.0261 (13)	0.0228 (13)	0.0040 (12)	0.0070 (12)	0.0036 (12)
C13	0.0271 (16)	0.052 (2)	0.049 (2)	0.0059 (15)	−0.0021 (15)	−0.0039 (17)
C14	0.0356 (19)	0.075 (3)	0.065 (3)	−0.0094 (19)	0.0131 (19)	0.005 (2)
C15	0.0310 (16)	0.0314 (16)	0.0377 (16)	−0.0018 (13)	−0.0020 (13)	−0.0010 (13)
C16	0.050 (2)	0.048 (2)	0.046 (2)	0.0013 (18)	−0.0098 (18)	−0.0121 (17)
C17	0.048 (2)	0.052 (2)	0.058 (2)	0.0180 (19)	0.0010 (18)	0.0167 (19)
C18	0.047 (2)	0.083 (3)	0.047 (2)	0.017 (2)	−0.0052 (19)	0.021 (2)
C19	0.065 (2)	0.0273 (16)	0.052 (2)	0.0037 (17)	0.012 (2)	−0.0016 (16)
C20	0.097 (4)	0.036 (2)	0.069 (3)	−0.011 (2)	0.008 (3)	0.0147 (19)
N1	0.0466 (16)	0.0240 (12)	0.0306 (14)	−0.0041 (11)	−0.0069 (12)	0.0060 (11)
N2	0.0334 (13)	0.0315 (13)	0.0263 (12)	0.0000 (11)	−0.0033 (10)	0.0040 (11)
N3	0.0217 (12)	0.0288 (12)	0.0342 (13)	0.0015 (10)	−0.0012 (10)	0.0016 (11)
N4	0.0410 (15)	0.0299 (13)	0.0407 (16)	0.0100 (12)	0.0044 (13)	0.0078 (12)
O1	0.0625 (17)	0.0525 (15)	0.0543 (16)	0.0216 (14)	0.0191 (14)	0.0003 (13)
O2	0.0563 (19)	0.143 (3)	0.084 (2)	0.002 (2)	−0.0089 (17)	0.042 (2)
O3	0.143 (3)	0.0545 (17)	0.078 (2)	0.049 (2)	0.012 (3)	0.0031 (17)
O4	0.0634 (19)	0.102 (2)	0.0473 (16)	0.0145 (18)	0.0108 (14)	0.0237 (17)
O5	0.093 (2)	0.0591 (16)	0.0472 (15)	−0.0381 (17)	−0.0234 (16)	0.0195 (13)
O6	0.0643 (19)	0.0660 (17)	0.094 (2)	0.0070 (16)	−0.0421 (19)	0.0015 (18)
O7	0.066 (2)	0.096 (2)	0.102 (3)	0.0200 (18)	−0.016 (2)	−0.057 (2)
O8	0.071 (2)	0.086 (2)	0.0544 (16)	−0.0347 (18)	−0.0061 (15)	0.0210 (16)
Cl1	0.0517 (5)	0.0519 (5)	0.0434 (5)	0.0224 (4)	0.0125 (4)	0.0125 (4)
Cl2	0.0420 (4)	0.0345 (4)	0.0402 (4)	−0.0056 (3)	−0.0080 (3)	0.0004 (3)
S1	0.0269 (3)	0.0302 (3)	0.0263 (3)	0.0038 (3)	−0.0007 (3)	0.0006 (3)
S2	0.0430 (5)	0.0411 (4)	0.0380 (4)	0.0119 (4)	0.0069 (4)	0.0016 (4)

### Geometric parameters (Å, º)

**Table:**

C1—C2	1.512 (4)	C12—N3	1.333 (4)
C1—S2	1.615 (3)	C13—N3	1.476 (4)
C1—S1	1.729 (3)	C13—C14	1.503 (5)
C2—N2	1.316 (4)	C13—H13A	0.9900
C2—N1	1.328 (4)	C13—H13B	0.9900
C3—N1	1.483 (4)	C14—H14A	0.9800
C3—C4	1.504 (6)	C14—H14B	0.9800
C3—H3A	0.9900	C14—H14C	0.9800
C3—H3B	0.9900	C15—N3	1.474 (4)
C4—H4A	0.9800	C15—C16	1.512 (5)
C4—H4B	0.9800	C15—H15A	0.9900
C4—H4C	0.9800	C15—H15B	0.9900
C5—N1	1.483 (4)	C16—H16A	0.9800
C5—C6	1.506 (7)	C16—H16B	0.9800
C5—H5A	0.9900	C16—H16C	0.9800
C5—H5B	0.9900	C17—N4	1.483 (5)
C6—H6A	0.9800	C17—C18	1.494 (5)
C6—H6B	0.9800	C17—H17A	0.9900
C6—H6C	0.9800	C17—H17B	0.9900
C7—N2	1.483 (4)	C18—H18A	0.9800
C7—C8	1.511 (5)	C18—H18B	0.9800
C7—H7A	0.9900	C18—H18C	0.9800
C7—H7B	0.9900	C19—N4	1.477 (4)
C8—H8A	0.9800	C19—C20	1.503 (5)
C8—H8B	0.9800	C19—H19A	0.9900
C8—H8C	0.9800	C19—H19B	0.9900
C9—N2	1.495 (4)	C20—H20A	0.9800
C9—C10	1.518 (5)	C20—H20B	0.9800
C9—H9A	0.9900	C20—H20C	0.9800
C9—H9B	0.9900	Cl1—O3	1.420 (3)
C10—H10A	0.9800	Cl1—O1	1.423 (3)
C10—H10B	0.9800	Cl1—O2	1.432 (4)
C10—H10C	0.9800	Cl1—O4	1.439 (3)
C11—C12	1.523 (4)	Cl2—O6	1.415 (3)
C11—S1	1.794 (3)	Cl2—O5	1.420 (3)
C11—H11A	0.9900	Cl2—O8	1.420 (3)
C11—H11B	0.9900	Cl2—O7	1.422 (3)
C12—N4	1.326 (4)		
			
C2—C1—S2	121.8 (2)	H13A—C13—H13B	108.0
C2—C1—S1	109.1 (2)	C13—C14—H14A	109.5
S2—C1—S1	129.03 (18)	C13—C14—H14B	109.5
N2—C2—N1	124.3 (3)	H14A—C14—H14B	109.5
N2—C2—C1	117.8 (2)	C13—C14—H14C	109.5
N1—C2—C1	117.9 (3)	H14A—C14—H14C	109.5
N1—C3—C4	110.6 (3)	H14B—C14—H14C	109.5
N1—C3—H3A	109.5	N3—C15—C16	111.1 (3)
C4—C3—H3A	109.5	N3—C15—H15A	109.4
N1—C3—H3B	109.5	C16—C15—H15A	109.4
C4—C3—H3B	109.5	N3—C15—H15B	109.4
H3A—C3—H3B	108.1	C16—C15—H15B	109.4
C3—C4—H4A	109.5	H15A—C15—H15B	108.0
C3—C4—H4B	109.5	C15—C16—H16A	109.5
H4A—C4—H4B	109.5	C15—C16—H16B	109.5
C3—C4—H4C	109.5	H16A—C16—H16B	109.5
H4A—C4—H4C	109.5	C15—C16—H16C	109.5
H4B—C4—H4C	109.5	H16A—C16—H16C	109.5
N1—C5—C6	111.3 (3)	H16B—C16—H16C	109.5
N1—C5—H5A	109.4	N4—C17—C18	113.5 (3)
C6—C5—H5A	109.4	N4—C17—H17A	108.9
N1—C5—H5B	109.4	C18—C17—H17A	108.9
C6—C5—H5B	109.4	N4—C17—H17B	108.9
H5A—C5—H5B	108.0	C18—C17—H17B	108.9
C5—C6—H6A	109.5	H17A—C17—H17B	107.7
C5—C6—H6B	109.5	C17—C18—H18A	109.5
H6A—C6—H6B	109.5	C17—C18—H18B	109.5
C5—C6—H6C	109.5	H18A—C18—H18B	109.5
H6A—C6—H6C	109.5	C17—C18—H18C	109.5
H6B—C6—H6C	109.5	H18A—C18—H18C	109.5
N2—C7—C8	111.4 (3)	H18B—C18—H18C	109.5
N2—C7—H7A	109.3	N4—C19—C20	112.9 (3)
C8—C7—H7A	109.3	N4—C19—H19A	109.0
N2—C7—H7B	109.3	C20—C19—H19A	109.0
C8—C7—H7B	109.3	N4—C19—H19B	109.0
H7A—C7—H7B	108.0	C20—C19—H19B	109.0
C7—C8—H8A	109.5	H19A—C19—H19B	107.8
C7—C8—H8B	109.5	C19—C20—H20A	109.5
H8A—C8—H8B	109.5	C19—C20—H20B	109.5
C7—C8—H8C	109.5	H20A—C20—H20B	109.5
H8A—C8—H8C	109.5	C19—C20—H20C	109.5
H8B—C8—H8C	109.5	H20A—C20—H20C	109.5
N2—C9—C10	110.8 (3)	H20B—C20—H20C	109.5
N2—C9—H9A	109.5	O3—Cl1—O1	108.8 (2)
C10—C9—H9A	109.5	O3—Cl1—O2	110.2 (3)
N2—C9—H9B	109.5	O1—Cl1—O2	109.9 (2)
C10—C9—H9B	109.5	O3—Cl1—O4	110.6 (2)
H9A—C9—H9B	108.1	O1—Cl1—O4	108.10 (18)
C9—C10—H10A	109.5	O2—Cl1—O4	109.2 (2)
C9—C10—H10B	109.5	O6—Cl2—O5	111.35 (18)
H10A—C10—H10B	109.5	O6—Cl2—O8	110.0 (2)
C9—C10—H10C	109.5	O5—Cl2—O8	109.14 (17)
H10A—C10—H10C	109.5	O6—Cl2—O7	109.5 (2)
H10B—C10—H10C	109.5	O5—Cl2—O7	109.5 (2)
C12—C11—S1	119.01 (19)	O8—Cl2—O7	107.2 (2)
C12—C11—H11A	107.6	C2—N1—C5	123.8 (3)
S1—C11—H11A	107.6	C2—N1—C3	120.5 (3)
C12—C11—H11B	107.6	C5—N1—C3	115.5 (3)
S1—C11—H11B	107.6	C2—N2—C7	124.9 (3)
H11A—C11—H11B	107.0	C2—N2—C9	120.8 (3)
N4—C12—N3	122.4 (3)	C7—N2—C9	114.1 (3)
N4—C12—C11	116.4 (3)	C12—N3—C15	122.9 (2)
N3—C12—C11	121.1 (2)	C12—N3—C13	119.4 (3)
N3—C13—C14	111.1 (3)	C15—N3—C13	117.7 (2)
N3—C13—H13A	109.4	C12—N4—C19	123.9 (3)
C14—C13—H13A	109.4	C12—N4—C17	122.4 (3)
N3—C13—H13B	109.4	C19—N4—C17	113.4 (3)
C14—C13—H13B	109.4	C1—S1—C11	104.50 (14)
			
S2—C1—C2—N2	−73.0 (3)	C10—C9—N2—C7	72.0 (4)
S1—C1—C2—N2	107.7 (3)	N4—C12—N3—C15	148.3 (3)
S2—C1—C2—N1	108.1 (3)	C11—C12—N3—C15	−27.5 (4)
S1—C1—C2—N1	−71.2 (3)	N4—C12—N3—C13	−33.0 (4)
S1—C11—C12—N4	146.7 (2)	C11—C12—N3—C13	151.1 (3)
S1—C11—C12—N3	−37.2 (4)	C16—C15—N3—C12	−122.3 (3)
N2—C2—N1—C5	−27.7 (5)	C16—C15—N3—C13	59.0 (4)
C1—C2—N1—C5	151.2 (3)	C14—C13—N3—C12	−127.1 (3)
N2—C2—N1—C3	157.5 (3)	C14—C13—N3—C15	51.6 (4)
C1—C2—N1—C3	−23.6 (4)	N3—C12—N4—C19	155.1 (3)
C6—C5—N1—C2	−57.2 (5)	C11—C12—N4—C19	−28.8 (4)
C6—C5—N1—C3	117.8 (4)	N3—C12—N4—C17	−31.9 (4)
C4—C3—N1—C2	−103.8 (4)	C11—C12—N4—C17	144.2 (3)
C4—C3—N1—C5	81.1 (5)	C20—C19—N4—C12	100.6 (4)
N1—C2—N2—C7	−26.8 (5)	C20—C19—N4—C17	−72.9 (4)
C1—C2—N2—C7	154.3 (3)	C18—C17—N4—C12	−51.3 (5)
N1—C2—N2—C9	157.3 (3)	C18—C17—N4—C19	122.4 (4)
C1—C2—N2—C9	−21.6 (4)	C2—C1—S1—C11	−169.24 (19)
C8—C7—N2—C2	−52.6 (4)	S2—C1—S1—C11	11.5 (3)
C8—C7—N2—C9	123.5 (3)	C12—C11—S1—C1	−67.1 (3)
C10—C9—N2—C2	−111.7 (4)		

### Hydrogen-bond geometry (Å, º)

**Table:**

*D*—H···*A*	*D*—H	H···*A*	*D*···*A*	*D*—H···*A*
C14—H14*B*···S1^i^	0.98	2.91	3.893 (4)	177

Symmetry code: (i) *x*−1, *y*, *z*.
